# Management of severe complications following penile surgery for erectile dysfunction and Peyronie disease: Three case reports

**DOI:** 10.1097/MD.0000000000018690

**Published:** 2020-02-14

**Authors:** Carlo Bettocchi, Valeria Santoro, Francesco Sebastiani, Giuseppe Lucarelli, Fulvio Colombo, David John Ralph, Mohamad Habous, Pasquale Ditonno, Michele Battaglia, Marco Spilotros

**Affiliations:** a Division of Urology, Department of Emergency and Organ Transplantation, University of Bari, Bari; b Department of Urology, Sant’Orsola Hospital - University of Bologna, Bologna, Italy; c The Institute of Urology, University College London Hospitals, London, UK; d Department of Urology and Andrology, Elaj Medical Center, Jeddah, Saudi Arabia.

**Keywords:** complications, erectile dysfunction, penile prosthesis, penile surgery, Peyronie disease, sliding technique

## Abstract

**Rationale:**

Erectile dysfunction (ED) and Peyronie's disease (PD) are conditions commonly observed in andrology. Despite the surgical refinement and the technical improvement in this field, even in expert hands, detrimental consequences have been reported and it can be related to patient's comorbidities or misconduct in the postoperative period. In this article we report anecdotal cases of severe complications following penile surgery for ED and PD in high volume centers, describe the strategies adopted to treat it and discuss the options that would have helped preventing these events.

**Patients’ concerns:**

The first case describes a patient with history of ED and PD causing penile shortening and a slight dorsal deviation of penile shaft. In the second case it is described a corporeal necrosis and urethral fistula following inflatable penile prosthesis implant. In the last case it is described the migration of reservoir into the abdomen after inflatable penile prosthesis implantation post-radical prostatectomy.

**Diagnosis:**

All 3 patients were investigated with a penile doppler ultrasound with PGE1 intracorporeal injection for ED and PD diagnosis. An abdominal computed tomography scan and magnetic resonance imaging were ordered for patient of case three.

**Interventions:**

The patients underwent different combined procedures depending on the case and including: glansectomy, penile prosthesis implantation associated with a penile elongation with double dorsal-ventral patch graft (“sliding technique”), penile urethroplasty with buccal mucosa graft, and laparotomy for reservoir removal.

**Outcomes:**

No further serious complications were reported after the procedures described.

**Lessons:**

Penile surgery in patients with concomitant PD and systemic comorbidities can be at high risk of complications. As shown in this series there are possible dramatic evolution of these complications that may cause irreversible consequences to the patient. For this reason, a dedicated surgical and nursing team is necessary to reduce the chances that it happens. When this event occurs, a team trained in their management can improve the patient outcome

## 1 Introduction

Erectile dysfunction (ED) and Peyronie's disease (PD) are conditions commonly observed in andrology and their prevalence can reach the 52% and 3% to 9% respectively.^[1–3]^ Medical approaches with oral tablets, intracorporeal injections, and local treatments to reduce the size of the plaque and the consequent penile curvature are largely used in patients who are not candidate for surgery.

Penile prosthesis implantation and plaque surgery for those cases who failed or are not fit for the available medical treatments requires significant skills in terms of technical approach to achieve satisfactory outcomes and a high level of expertise to manage the potentially dramatic complications that can be observed. For this reason, nowadays, these conditions are investigated and treated mainly in high volume centers where the chances of positive results with minimal side effects can be achieved. Contextually great efforts have been practiced by the main penile prosthesis manufacturers since their commercialization to improve the mechanical reliability and to reduce the rate of the most concerning complication, infection.^[4,5]^ Standardization of the technique and the careful attention to prevent the contact between the implant and potential contaminant represents a further step towards results optimization and reduction of complications.^[6]^ As result the couple satisfaction following penile prosthesis implant remains high.^[7,8]^ PD is responsible of anatomic changes in the penile corporas including plaques, hourglass deformity, penile shortening and in the most severe cases it can be associated to ED.^[9–11]^ From a surgical point of view three main approaches are described: plication of the corpora for curvatures <60°; grafting techniques using autologous or non-autologous graft for curvatures >60°; penile prosthesis implantation and corporoplasty in patient with penile curvature and ED not responding to medical therapy.^[9]^ A combined approach using grafting with a “sliding technique” and inflatable penile implant insertion for those patients with significant shortening and ED have been reported and is now recognized as one of the possible surgical option.^[12]^

Despite the surgical refinement and the technical improvement in this field, even in expert hands, detrimental consequences have been reported and it can be related to patient's comorbidities or misconduct in the post-operative period, wound infection, mechanical failure and technical imperfection or misjudgments during the procedure.

Our aim is to report anecdotal cases of severe complications following penile surgery for ED and PD in high volume centers, describe the strategies adopted to treat it and discuss the options that would have helped preventing these events.

All patients provided informed consent for publication of the cases.

## 2 Case reports

### 2.1 Case 1: sub-total glansectomy following penile implant insertion in insulin dependent diabetic patient

A 51 years-old patient suffering from type II diabetes mellitus (DM) on insulin treatment, severe obesity (body mass index = 37) and coronary heart disease presented to our attention complaining of a ten years history of ED and PD causing penile shortening and a slight dorsal deviation of penile shaft observed in those occasions when a partial tumescence occurred. Previous treatment with different PDE5i and intracorporeal injection of Alprostadil at progressively higher doses did not provide satisfactory results.

The physical examination revealed a stretched penile length of 10 cm and multiple fibrotic plaques along the whole length of the dorsal aspect of the penile shaft. The patient was further investigated with a Penile Doppler US with PGE1 intracorporeal injection showing a poor arterial inflow (peak systolic velocity = 19 cm/s) consistent with his comorbidities and a clinical response of 3/5 (partial tumescence not sufficient for penetration). Considering the poor response reported to conservative treatments, the patient was counseled for a penile prosthesis insertion +/− grafting. After a demonstration of the available devices in clinic the patient choice was for an inflatable device.

The surgical procedure was performed by an expert surgeon through a peno-scrotal approach. The preoperative management consisted of i.v. Cefamezin 1 g/10 mL at induction, a careful shaving of the surgical area in the operating room and a preoperative Hibiscrub wash of the genitalia and lower abdomen performed for 10 minutes. The isolation of the corporas and the access to the Retzius space considering that the patient was surgery-naïve was uncomplicated. Corporotomies and the following dilatation were more laborious due to the significant amount of fibrosis encountered: this procedure was performed using Metzenbaum scissors and Hegar dilators without needing additional tools or longer corporotomies for fibrotic corporas. Cross-over, corporal performation, and urethral erosion were excluded before the penile prosthesis insertion. After measurement of the corporas 15 + 1 cm cylinders (AMS 700 CX) were implanted, the MS pump was positioned in the scrotum and the 75 mL Reservoir was inserted in the left Retzius space. A minimal residual dorsal curvature (<30^o^) was corrected with a modeling maneuver and did not require the additional use of a graft.^[13]^ A “mummy wrap” was applied to the scrotum and penile shaft. The prosthesis was left 70% inflated for 24 hours.

The first postoperative day the dressing was removed revealing no signs of hematoma or suspicious swelling; the device was completely deflated and the patient discharged with a 7-days course of oral Co-amoxiclav. Strong recommendation regarding strict interdiction to sexual intercourse for 6 weeks was reiterated to the patient. An outpatient appointment in two weeks was arranged for review.

**Figure 1:**
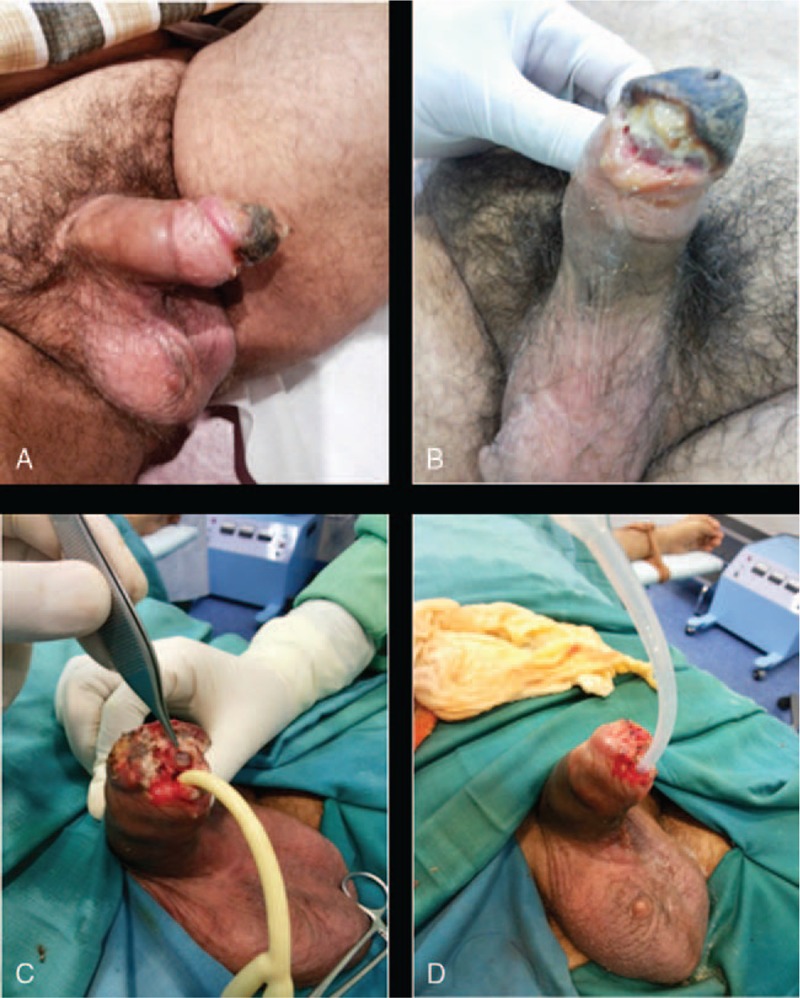
A dusky area on the dorso-lateral portion of the glans and the penile prosthesis inflated (A); The glans was completely necrotic and the left cylinder was eroded from the distal portion of the corpora (B). The left cylinder was eroded through the corpora and the lateral aspect of the distal urethra which was clearly infected (C). The urethra was excised distally up to the level where healthy spongiosa was seen, spatulated ventrally and anchored to the surviving portion of the glans (D).

The first postoperative check in clinic demonstrated a dusky area on the dorso-lateral aspect of the glans penis and the penile prosthesis inflated. A careful examination didn’t show cylinder erosion nor swelling of the shaft and scrotum (Fig. 1A). The patient admitted that he had two different sexual intercourses with his partner starting 1 week postoperatively against the recommendation of the clinicians. A conservative approach was adopted: the implant was deflated promptly and a regular wound medication using betadine solution and topical gentamicin was performed. The patient was reviewed on a regular basis in clinic for the following four weeks. At 6 weeks post-surgery the situation appeared dramatic and not salvable with conservative treatments. As shown in Figure 1B, the glans penis was completely necrotic and the distal portion of the left corpora was perforated by the distal cylinder. Purulent discharge coming out from the sulcus was evident and the patient reported voiding difficulties. The proximal aspect of the shaft and the scrotum appeared intact without signs of cellulitis or swelling. The ultrasound of the penis demonstrated an intact blood supply to the distal penile shaft and a narrowing of the urethra up to 1 cm below the glans secondary to dense fibrotic reaction. Surgical review was recommended to the patient and he was counseled about glansectomy with meatoplasty and prosthesis removal.

The procedure was performed 3 months after the original procedure of penile prosthesis insertion. Glans debridement was performed and a sub-total glansectomy was deemed necessary due to the large amount of necrotic tissue. The left cylinder was clearly eroded through the corpora and the lateral aspect of the distal urethra and it was clearly infected (Fig. 1C). A re-do peno-scrotal incision was then performed to remove the whole device including pump and reservoir and a Mulchay salvage procedure was performed to wash-out the area and minimalizing the risks of infection spread.^[14]^ The urethra was excised distally up to the level where healthy spongiosa was seen, spatulated ventrally and anchored to the surviving portion of the glans with interrupted resorbable sutures on an 18 Fr Foley catheter (Fig. 1D). The patient was discharged 4 days postoperatively with a 7 days course of oral penicillin and he was reviewed weekly in clinic for wound check. He was started on vacuum therapy as soon as the external wound healing was documented and the catheter removed to reduce corporeal fibrosis in consideration of re-do penile implant insertion.

The follow-up was uneventful and a complete healing of the external meatus and the remaining glans was observed. The regular use of the vacuum device provided an excellent result in terms of minimal scarring of the penile shaft. Six months after the last surgical procedure the patient underwent to a penile implant insertion (AMS 700 CX) through a peno-scrotal incision performed by the same surgeon. The long use of the vacuum therapy made the dilatation of the corpora uncomplicated.

The standard postoperative care used in occasion of the first implant was adopted and the patient followed carefully our recommendation and avoided sexual intercourses for 6 weeks after surgery. After 18 months of follow up no complications have been recorded and the patient is using regularly the device reporting satisfactory results.

### 2.2 Case report 2: corporeal necrosis and urethral fistula following inflatable penile prosthesis implant and “sliding technique”

A 51 years old man with a 5 years history of mild ED and Peyronie disease presented to our attention. He reported poor response to PDE5i and a stable plaque along the lateral aspect of the penis for the previous 12 months. He did not present significant comorbidities but reported a significant alcohol consumption and smoked 20 cigarettes per day. He underwent circumcision in childhood. A penile Doppler US showed arterial deficiency (peak systolic velocity = 20 cm/s) and a 25 × 17 mm calcified plaque of the mid-shaft causing significant penile shortening and a slight lateral deviation of about 45^o^. The patient was counseled regarding the possibility of a combined procedure to improve his erection and restore penile length and deviation. The “sliding technique” offered to the patient previously described by Rolle et al^[12]^ consisted of a penile prosthesis implantation together with a penile elongation thanks to a double dorsal-ventral patch graft. The aim of this surgical choice was both to restore the length lost due to PD and to guarantee the rigidity necessary to engage in penetrative sexual intercourse. The patient was fully aware about the complexity and the potential risk associated with the procedure, which is quite complex when compared to prosthesis implantation alone. Considering the severe level of distress caused by his double condition, the patient choice was to have the surgery recommended.

**Figure 2:**
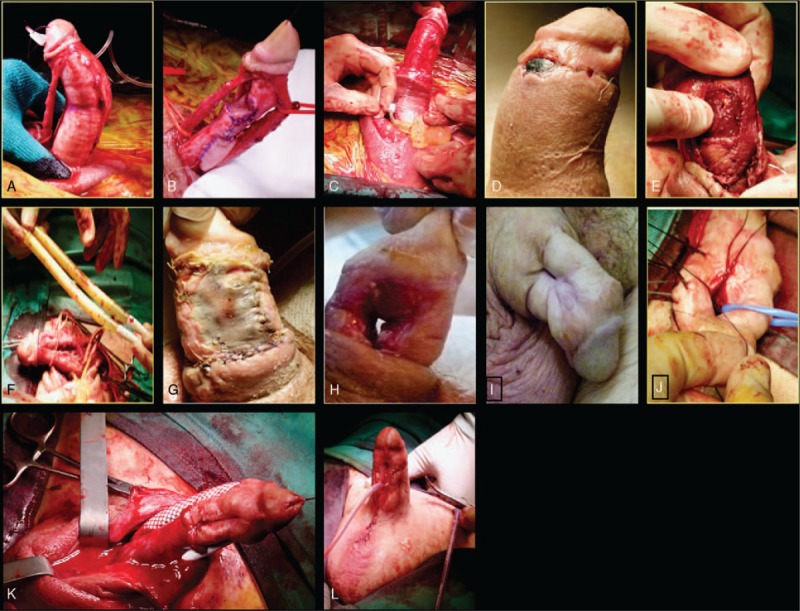
The surgical technique was conducted using a combined penoscrotal and subcoronal incision followed by a complete degloving of the penile shaft. NVB and urethra were completely mobilized from the underlying tunica albuginea to guarantee maximum lengthening (A). The sliding of the 2 segments of the shaft led to the formation of 2 rectangular tunical defects on opposite sides of the shaft penis, which were covered with an autologus graft (B). The cylinders of an inflatable 3-pieces penile prosthesis were then implanted through the ventral albugineal defect; an adequate straightening of the shaft showed intraoperatively a 3 cm lengthening of the penile shaft (C). Two weeks postoperatively the patient complain pain of the distal shaft associated with a dusky area along the sub-glandular suture line and purulent discharge coming out from the wound (D). The distal penile shaft appeared ischemic and a small defect of the lateral aspect of the right corpora that was elongated during the first stage was observed (E). The implant was explanted to improve the blood supply (F). Necrosis of the full-thickness skin graft used to recreate a decent thickness penile skin without tension (G). The progressive necrosis of the area involving the proximal part of the penile urethra resulting in a large fistula (H). Final appearance of the penis 2 months after surgery (I). First stage urethroplasty: an adequate urethral plate was created using a BMG quilted onto the remaining albuginea of the corpora with interrupted sutures (J). Malleable penile implant covered in 2 dacron sleeves to reinforce the corpora cavernosa extremely flimsy and partially necrotic (K). Final appearance of the refashion penis after reconstructive surgery (L). BMG = buccal mucosa graft, NVB = neurovascular bundle.

The surgical technique was conducted using a combined penoscrotal and subcoronal incision followed by a complete degloving of the penile shaft. Buck's fascia was incised and the neurovascular bundle (NVB) completely mobilized from the tunica albuginea down to the origin of the suspensory ligament to maximize lengthening. In case of sliding technique, the length of the NVB represents the limiting factor in the lengthening process because it cannot be stretched excessively to avoid blood supply compromising. The urethra was then dissected off the corpora cavernosa and the penis partially disassembled (Fig. 2A). Two longitudinal incisions of the tunica albuginea of about 4 cm in length were then made and the edges of these 2 incisions were then joined by 2 semi-circumferential transverse incisions. The proximal transverse incision was made on the ventral side of the penis at the level of the penoscrotal junction, to allow the insertion of the cylinders and connecting tubing of the inflatable penile prosthesis, avoiding in this way the need to make a second proximal ventral corporotomy, while the distal incision was carried out on the dorsal side of the shaft. This manouvre, transecting the corporas, literally led to the sliding of the distal portion away from the proximal aspect of the shaft along the two previously performed longitudinal incisions. At this stage, the maximum elongation of the NVB indicates how much the two sections of the shaft could be slid apart, as the spongiosum of the urethra could elongate significantly more than the NVB. When the maximum tension on the NVB and the urethra was obtained, the two segments of the shaft were fixed laterally along the two longitudinal tunical incisions with resorbable sutures. The sliding of the 2 segments of the shaft led to the formation of 2 rectangular tunical defects on opposite sides of the shaft penis, which were covered with an autologus graft (Fig. 2B).

The cylinders of an inflatable penile prosthesis were then implanted through the ventral albugineal defect; the cylinders were left semi-inflated to reduce the risk of hematoma and to allow the formation of a capsule around the cylinders. An adequate straightening of the shaft was documented intraoperatively with a 3 cm lengthening of the penile shaft (Fig. 2C). A compressive dressing was then applied to the scrotum and to the penile shaft and left in situ for 3 days. The patient was discharged 3 days postoperatively with oral antibiotics and was instructed regarding wound care.

Two weeks postoperatively the patient attended the outpatient appointment complaining pain of the distal shaft associated with a dusky area along the sub-glandular suture line and purulent discharge coming out from the wound (Fig. 2D). The skin of the penile shaft was under clear tension and it could have been a concurrent factor implicated in the distal necrosis. At this stage the decision to surgically review the wound was made.

The patient was admitted to hospital for an elective review. Preoperative broad-spectrum antibiotics were given at induction and prolonged for 7 days postoperatively. The distal penile shaft appeared ischemic and a small defect of the lateral aspect of the right corpora that was elongated during the first stage was observed (Fig. 2E and F). Considering the high risk of worsening of the necrotic damage, the implant was explanted to improve the blood supply, the defect on the corpora was repaired and overlapped with the dartos available (Fig. 2G). The main limiting aspect for the successful closure of the wound was the small amount of penile skin available. For this reason a full-thickness skin graft was harvested from the lower abdomen and used to recreate a decent thickness penile skin without tension. A gentle compressive dressing of the shaft was left in situ for 7 days.

The following outpatient appointments arrange twice-weekly revealed a poor graft adhesion on the ventral aspect of the penile shaft and, at a later stage, a progressive necrosis of the area involving the proximal part of the penile urethra resulting in a large fistula (Fig. 2H and I).

Two months later the patient underwent to a first stage penile urethroplasty and malleable penile implant covered in 2 dacron sleeves to reinforce the corpora cavernosa extremely flimsy and partially necrotic. An adequate urethral plate was created using a buccal mucosa graft quilted onto the remaining albuginea of the corpora with interrupted sutures (Fig. 2J). The corporas were reconstituted using two dacron grafts surrounding the malleable rods (Fig. 2K). A tension-free closure in multiple layers was performed at the end of the procedure. The patient was discharged three days after surgery with a compressive dressing and an indwelling catheter, both removed 2 weeks later (Fig. 2L). At 3 months of follow-up no early postoperative complications have been recorded with a good health of the penile shaft and the urethral plate. The second-stage urethroplasty was scheduled 6 months after the last procedure.

### 2.3 Case 3: migrating reservoir into the abdomen after inflatable penile prosthesis post-radical prostatectomy

A 54 years old man presented with ED secondary to a robotic radical prostatectomy in 2005. He had not responded to PDE5i inhibitors, intracavernosal injection therapy with PGE1 or vacuum therapy. He had a history of hypertension, diabetes, and chronic renal failure, all of which were well controlled. He also was incontinent, needing 4 pads per day and so initially underwent the placement of an artificial urinary sphincter (AUS) in 2008. This proceeded uneventfully with the balloon of the AUS placed in the right iliac fossa.

**Figure 3:**
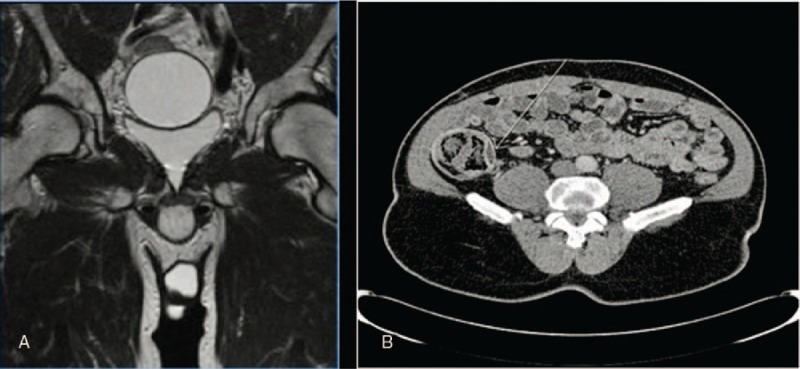
MRI showed the reservoir to be full and centrally placed within the pelvis (A). Abdominal CT scan revealed that the reservoir had migrated into the abdomen and had become wrapped around the caecum (B). CT = computed tomography, MRI = magnetic resonance imaging.

Two years later he had the insertion of an inflatable penile prosthesis (AMS 700 CX) using a 100 mL reservoir placed in the left retropubic space using a blind puncture through the external ring. He made an unremarkable post-operative recovery and was discharged with the implant deflated to be reviewed in clinic. Over the following 3 months he had problems inflating the device due to a “sticky pump” and pain in the right iliac fossa. To exclude a device leak an magnetic resonance imaging was ordered which showed the reservoir to be full and centrally placed within the pelvis (Fig. 3A).

The patient was then offered an exchange of pump but decided instead to have the device replaced with a malleable penile implant which was performed after an additional 3 months. The cylinders and pump were removed and a malleable Genesis implant inserted after a mini salvage washout. The reservoir was emptied and retained after the tubing was cut high. At follow up the malleable implant was excellently sited but the patient continued to experience right abdominal pain. An abdominal CT scan was ordered which revealed that the reservoir had migrated into the abdomen and had become wrapped around the caecum (Fig. 3B). Removal of the reservoir was planned initially by a laparoscopic approach although due to adhesions it was difficult to identify it. An open incision was then made in the right iliac fossa but the reservoir could not be found. A second midline upper abdominal incision was made and the reservoir retrieved for the left upper quadrant. A speedy recovery was made, the right abdominal pain resolved and the patient was happily having sexual intercourse.

## 3 Discussion

The first case report describes a severe complication resulting in glans necrosis following inflatable penile prosthesis insertion in a patient with corporal fibrosis and several comorbidities. It is always debating the correct timing of surgical revision in case of initial signs of infection. The first patient; however, had a type II DM on insulin treatment with severe obesity, in other terms a patient with higher risks of infection. The initial signs of dusky skin associated with the history of premature use of the device were a clear alarm that he was developing an ischemic process often associated to infection in this population. A long observation with conservative treatments could delay an active intervention and in this case resulted in the need to debride the glans and refashion the distal urethra. A closer control or a prompt removal of the penile prosthesis in this scenario could have resulted in a less dramatic complication.

Patients with Peyronie disease and ED often require penile augmentation to restore the original length lost due to large penile fibrosis and retraction. The sliding technique described in the second case report is a possible surgical option to offer although it is a complex procedure with high risk of complications. The NVB and urethra stretching may end up with tissue ischemia and necrosis, if the final tension is excessive. It is difficult sometimes to judge correctly how much you can stretch the tissues and so you may see post-operatively the appearance of necrotic areas that need to be removed and treated releasing the present tension. This case is very unfortunate because although the surgeon decided a prompt surgical action with the removal of the entire device to avoid any possible tension, then the graft did not take to the underlying unhealthy tissue and ended up with a large necrosis and loss of part of the corpora cavernosa. The lessons learnt from this complication are to reduce as much as possible the tension along the corpora in case of sliding technique to increase the blood supply to the penis minimizing the possibility of necrosis of the surrounding structures. In the last case report we have described a significant migration into the abdomen of an inflatable penile prosthesis reservoir. The consideration that needs to be done following this complication is that retropubic reservoir insertion following a robotic prostatectomy is likely to be intraperitoneal due to the taking down of the pelvic peritoneum to gain access to the prostate. A separate incision in the lower abdomen should be preferred to reach the extraperitoneal space avoiding migration into the abdomen. Imaging should be performed either immediately before the operation or ideally intraoperatively to locate the current position the reservoir and to avoid losing it and having to blindly search with the wrong incision.

## 4 Conclusion

Penile surgery in patients with concomitant PD and systemic comorbidities can be at high risk of complications. As shown in this series there are possible dramatic evolution of these complications that may cause irreversible consequences to the patient. For this reason, a dedicated surgical and nursing team is necessary to reduce the chances that it happens. When this event occurs, a team trained in their management can improve the patient outcome.^[15]^

## Author contributions

**Conceptualization:** Carlo Bettocchi.

**Data curation:** Carlo Bettocchi, Valeria Santoro, Francesco Sebastiani, Giuseppe Lucarelli, Fulvio Colombo, Mohamad Habous, Pasquale Ditonno, Michele Battaglia, Marco Spilotros.

**Investigation:** Carlo Bettocchi, Francesco Sebastiani, Giuseppe Lucarelli, Fulvio Colombo, Mohamad Habous, Pasquale Ditonno, Michele Battaglia.

**Methodology:** Carlo Bettocchi.

**Supervision:** Carlo Bettocchi, David J. Ralph, Marco Spilotros.

**Writing – original draft:** Carlo Bettocchi, Valeria Santoro, Marco Spilotros.

**Writing – review and editing:** Giuseppe Lucarelli.

Giuseppe Lucarelli orcid: 0000-0001-7807-1229.
